# Molecular characterization of cell-free eccDNAs in human plasma

**DOI:** 10.1038/s41598-017-11368-w

**Published:** 2017-09-08

**Authors:** Jing Zhu, Fan Zhang, Meijun Du, Peng Zhang, Songbin Fu, Liang Wang

**Affiliations:** 1 Laboratory of Medical Genetics, Harbin Medical University, Harbin, Heilongjiang 150081 China; 2 Department of Pathology and MCW Cancer Center, Medical College of Wisconsin, Milwaukee, WI 53226 USA; 3 School of Computer Science and Technology, Harbin Institute of Technology, Harbin, Heilongjiang 150001 China

## Abstract

Extrachromosomal circular DNAs (eccDNAs) have been reported in most eukaryotes. However, little is known about the cell-free eccDNA profiles in circulating system such as blood. To characterize plasma cell-free eccDNAs, we performed sequencing analysis in 26 libraries from three blood donors and negative controls. We identified thousands of unique plasma eccDNAs in the three subjects. We observed proportional eccDNA increase with initial DNA input. The detected eccDNAs were also associated with circular DNA enrichment efficiency. Increasing the sequencing depth in an additional sample identified many more eccDNAs with highly heterogenous molecular structure. Size distribution of eccDNAs varied significantly from 31 bp to 19,989 bp. We found significantly higher GC content in smaller eccDNAs (<500 bp) than the larger ones (>500 bp) (p < 0.01). We also found an enrichment of eccDNAs at exons and 3′UTR (enrichment folds from 1.36 to 3.1) as well as the DNase hypersensitive sites (1.58–2.42 fold), H3K4Me1 (1.23–1.42 fold) and H3K27Ac (1.33–1.62 fold) marks. Junction sequence analysis suggested fundamental role of nonhomologous end joining mechanism during eccDNA formation. Further characterization of the extracellular eccDNAs in peripheral blood will facilitate understanding of their molecular mechanisms and potential clinical utilities.

## Introduction

Extrachromosomal DNA, also called extrachromosomal elements (EEs), is the DNA elements physically separated from the chromosomes, which reflects the plasticity of the genome and may be related to genome evolution and genomic disorders. A major form of extrachromosomal DNA is extrachromosomal circular DNA (eccDNA) which organizes itself in a closed circular form. eccDNA presents in all eukaryotic cells and can be classified as organelle eccDNA such as mitochondrial DNA, and more flexible non-organelle eccDNA such as episomes, small polydispersed circular DNA (spcDNA), double minute chromosomes (DMs) and microDNA in eukaryote cells^1–3^. An abundant number of eccDNAs in normal, aged or diseased cells have been reported. However, little is known about the extracellular eccDNAs that are released from cells into bloodstream (circulating cell free eccDNAs).

eccDNAs are a less studied form of genomic copy number variation (CNV). Their sizes are heterogeneous from hundreds of base pairs (bp) to as large as several mega bases (Mb). eccDNAs may be originated from genomic tandem repeats or unique gene regions^4, 5^. For example, as a vehicle of extrachromosomal gene amplification, DMs usually carry oncogenes or drug resistance genes to increase the gene dosage and provide growth advantage to tumor cells^6^. The newly discovered small eccDNAs, called microDNAs, are preferentially derived from exons, 5′ UTRs and CpG islands^3, 7^. On the other hand, tandemly repeated genomic sequences or interspersed repeat sequences have been reported as a predominant component of eccDNAs in Drosophila^8^, plant^9^ and animal cells^10^. Although elevated eccDNAs have been observed in tumor cells, drug resistant cells and aging cells, characterization of human circulating extracellular eccDNAs has not been reported.

Cell free nucleic acids are discovered in human plasma in the late 1940^11^. Since then, much emphasis has been placed on the potential of circulating nucleic acids in serum or plasma to serve as biomarkers for various human diseases^12, 13^. For example, the circulating cell free DNA (cfDNA), released from dead tumor cells into the bloodstream, is associated with tumor occurrence and treatment response, and is believed to be sensitive biomarkers for tumor diagnosis and prognosis through noninvasive liquid biopsy^14, 15^. Because eccDNAs are more stable than linear DNAs, the eccDNAs circulating in blood are an ideal source for potential biomarker exploration. In this study, we applied next generation sequencing (NGS) to profile the cell free eccDNAs in plasma and described the molecular characteristics, potential functional roles and possible formation mechanisms of the cell free eccDNAs.

## Results

### Sequencing libraries and their quality

**Figure 1:**
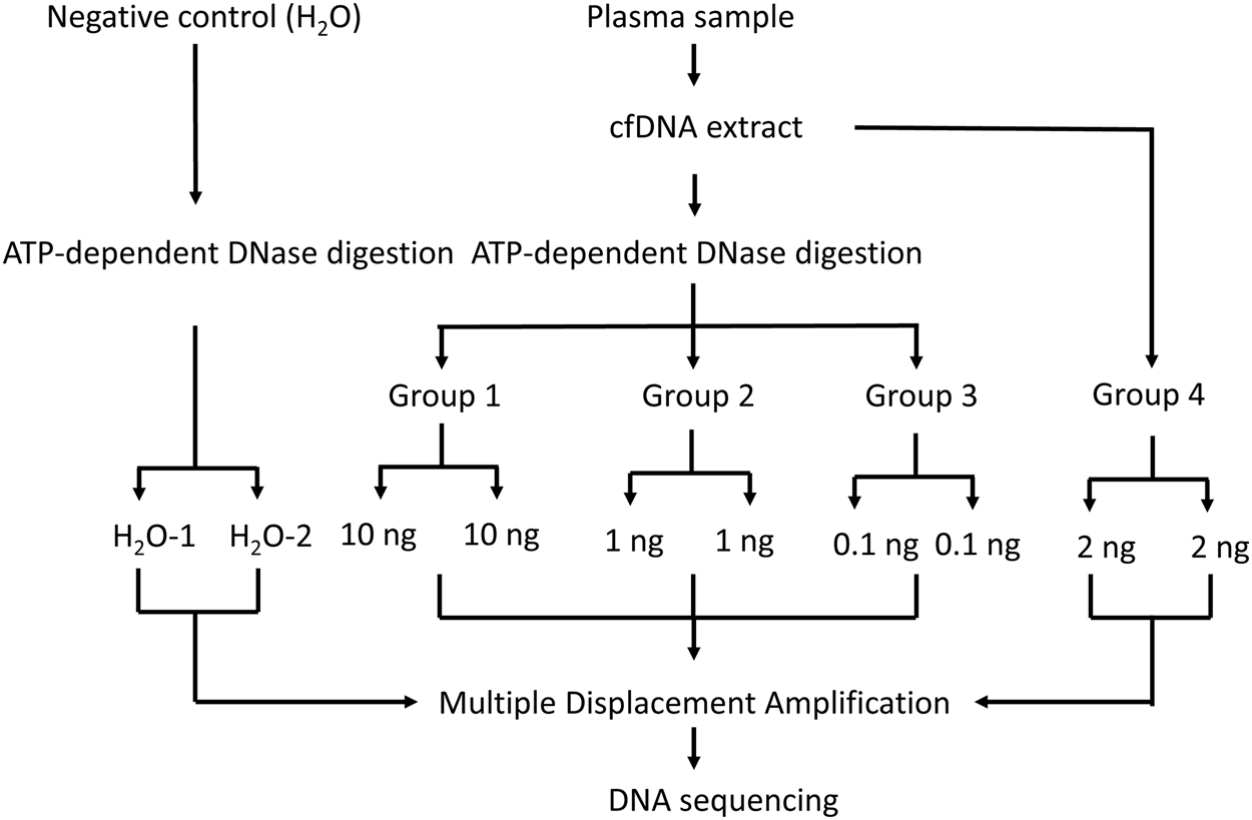
Study design to characterize eccDNAs in human plasma. cfDNAs from three plasma samples were divided into four groups. Groups 1, 2 and 3 were subjected to the ATP-dependent DNase digestion and MDA procedures while Group 4 went directly to the MDA procedure. Each group has a technical replicate. Water was used as a negative control. A total of 26 libraries were prepared for DNA sequencing. Another four libraries from one additional plasma cfDNA sample were sequenced as a validation.

**Figure 2:**
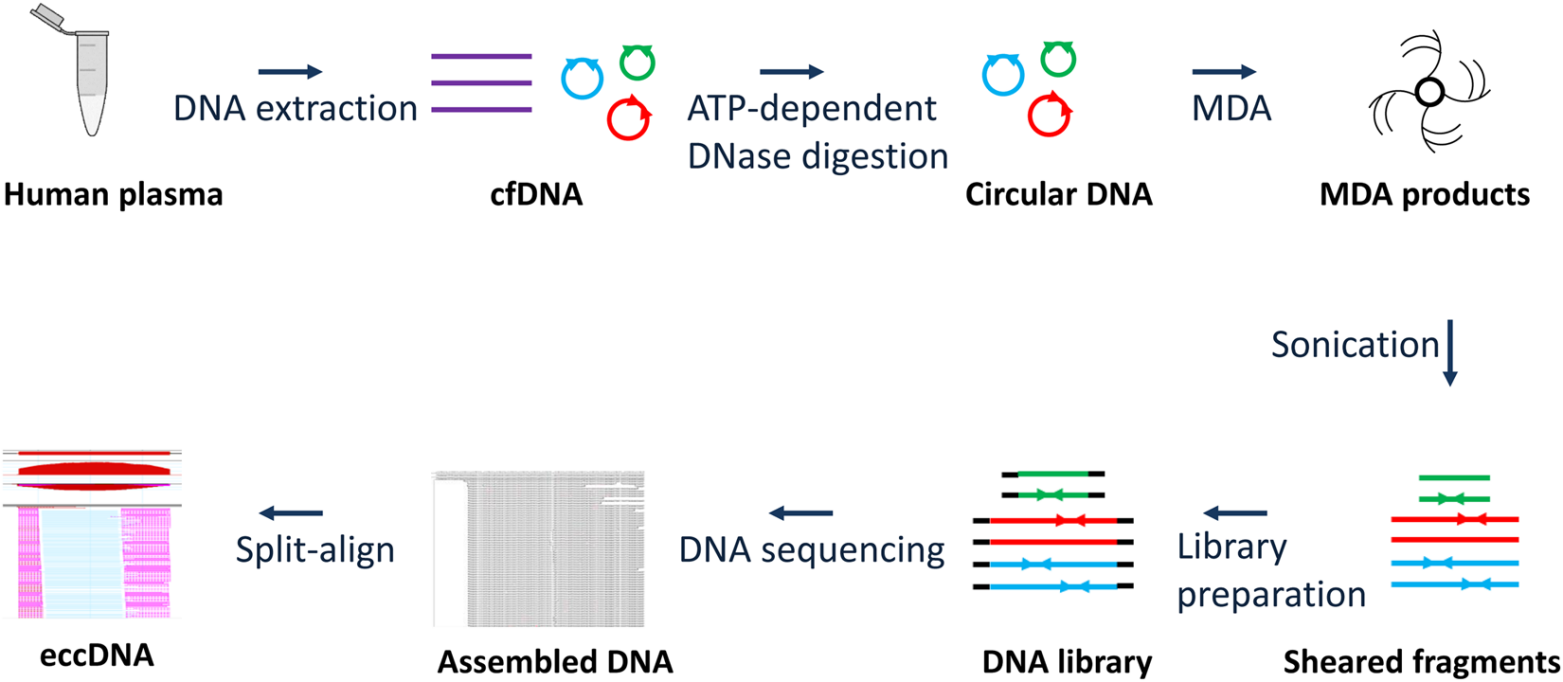
Flow chart of eccDNA preparation and sequencing data analysis. cfDNA was extracted from human plasma. Plasmid-Safe ATP-dependent DNase was used to remove the linear DNA. The DNase-resistant DNA (circular DNA) was further amplified using MDA method. The MDA products were sheared into 100–300 bp fragments, which were then used for library preparation and sequencing analyses. By removing mitochondrial sequence, the remaining sequences were aligned to human genome sequences. A split-read based method (Split-align) was used to determine the eccDNAs.

To enrich the eccDNAs, we treated the cfDNAs using ATP-dependent DNase which selectively digested linear DNA. We then applied the MDA method to preferentially amplify the remaining circular DNAs (Figs 1 and 2. See details in Methods). To demonstrate successful amplification, we ran the amplified DNAs on agarose gel and observed the products with average size >10 kb (Supplementary Fig. S1). After the amplified products were fragmented by sonication and sequencing libraries were prepared, we also ran the agarose gel and observed the DNA fragments with expected sizes (the sonicated DNA fragments sized 100–300 bp and the DNA libraries sized 250–500 bp) (Supplementary Fig. S2). Together, we prepared 26 sequencing libraries (13 technical replicates) from three cfDNA samples (Sample 1, 2 and 3) and negative control group (H_2_O groups) (Fig. 1). For each library (excluding H_2_O groups), we received ~13.2 million (8.02~21.19 million) sequence reads that were mapped to whole human genome (Table 1). In H_2_O groups, the raw read count was 9.0–10.8 million but the human genome-mapped sequences are only 0.06–0.86 million (Table 1). Absence of human genome-mapped sequences from H_2_O control indicated non-specific amplification of MDA procedure in H_2_O samples. In samples 1 and 2, the digested group had an average of 3.42–6.70 million reads of mitochondrial DNA, while the undigested group had 0.046–0.166 million reads of mitochondrial DNA. In sample 3, however, the mitochondrial DNA is 0.07–0.13 million in digested groups and 0.15–0.16 million in undigested groups (Table 1).

**Table 1:** Statistical summary of eccDNA sequencing data in samples 1, 2, 3 and negative control.

Sample name	Group name	Raw read count	Mapped sequence count	Mapped genomic (MT-removed)	Mapped MT DNA	Split read counts	Number of eccDNA	MT/genome (%)
Sample 1	Group1-1	15,313,740	12,892,368	8,987,862	3,904,506	28327	1023	30.3
	Group1-2	13,302,692	11,085,619	7,838,777	3,246,842	26914	1007	29.3
	Group2-1	12,262,866	10,169,673	7,539,565	2,630,108	16630	245	25.9
	Group2-2	15,852,854	14,194,811	8,595,658	5,599,153	14002	144	39.4
	Group3-1	14,201,004	12,081,204	9,392,788	2,688,416	21175	63	22.3
	Group3-2	12,860,924	10,965,787	8,488,530	2,477,257	17150	54	22.6
	Group4-1	12,822,504	12,398,042	12,350,579	47,463	9784	3	0.4
	Group4-2	14,064,008	13,607,707	13,563,040	44,667	11286	3	0.3
Sample 2	Group1-1	12,905,004	9,291,546	3,343,414	5,948,132	22422	533	64.0
	Group1-2	11,469,530	8,025,091	2,787,004	5,238,087	16545	496	65.3
	Group2-1	13,057,148	9,246,520	2,353,859	6,892,661	17229	247	74.5
	Group2-2	14,483,862	10,391,664	2,590,058	7,801,606	19750	251	75.1
	Group3-1	13,187,350	9,135,157	2,270,383	6,864,774	18041	69	75.1
	Group3-2	13,815,354	10,247,422	2,799,433	7,447,989	31804	55	72.7
	Group4-1	16,127,688	15,384,478	15,176,916	207,562	15814	9	1.3
	Group4-2	13,845,566	13,346,635	13,220,564	126,071	12249	9	0.9
Sample 3	Group1-1	22,470,872	21,192,169	21,080,441	111,728	12300	6	0.5
	Group1-2	15,418,760	14,619,395	14,544,657	74,738	8842	5	0.5
	Group2-1	18,691,408	16,934,676	16,799,835	134,841	9514	5	0.8
	Group2-2	17,412,528	16,034,045	15,933,392	100,653	10563	5	0.6
	Group3-1	15,618,252	14,672,650	14,579,255	93,395	13414	15	0.6
	Group3-2	16,886,270	15,667,528	15,553,053	114,475	11887	11	0.7
	Group4-1	20,241,308	18,727,546	18,578,536	149,010	10060	4	0.8
	Group4-2	18,388,708	17,196,472	17,037,181	159,291	10856	6	0.9
H_2_O	H_2_O -1	8,995,040	856,937	856,021	916	3509	5	0.1
	H_2_O -2	10,875,278	57,090	56,262	828	4	0	1.5

### Presence of eccDNAs in human plasma

By excluding sequence reads mapping to mitochondria, 2.3–21 million remaining sequences were used to identify the eccDNAs using split-align algorithm based on mapped read positions and directions of two split sequences. A typical eccDNA has split reads forming the sharp external boundaries and abundant continuous sequence reads within the boundaries. When split read depth ≥3 was used as a cutoff, we detected a total number of 2542, 1669, and 57 unique eccDNAs in samples 1,2 and 3 respectively (excluding the undigested groups). In contrast, the negative controls (H_2_O) showed < = 5 eccDNAs, indicating the specific enrichment of eccDNAs in tested samples (Table 1). To verify the eccDNAs from DNA sequencing results, we selected eccDNA targets with different read depth (read depth = 3~1384) and carried out junction-specific PCRs (Supplementary Table 1). Compared to normal genome DNA, only MDA-derived products generated specific bands with the expected sizes, confirming the presence of the eccDNAs in pre-library DNA samples (Supplementary Fig. S3).

### Factors affecting eccDNA identification

**Figure 3:**
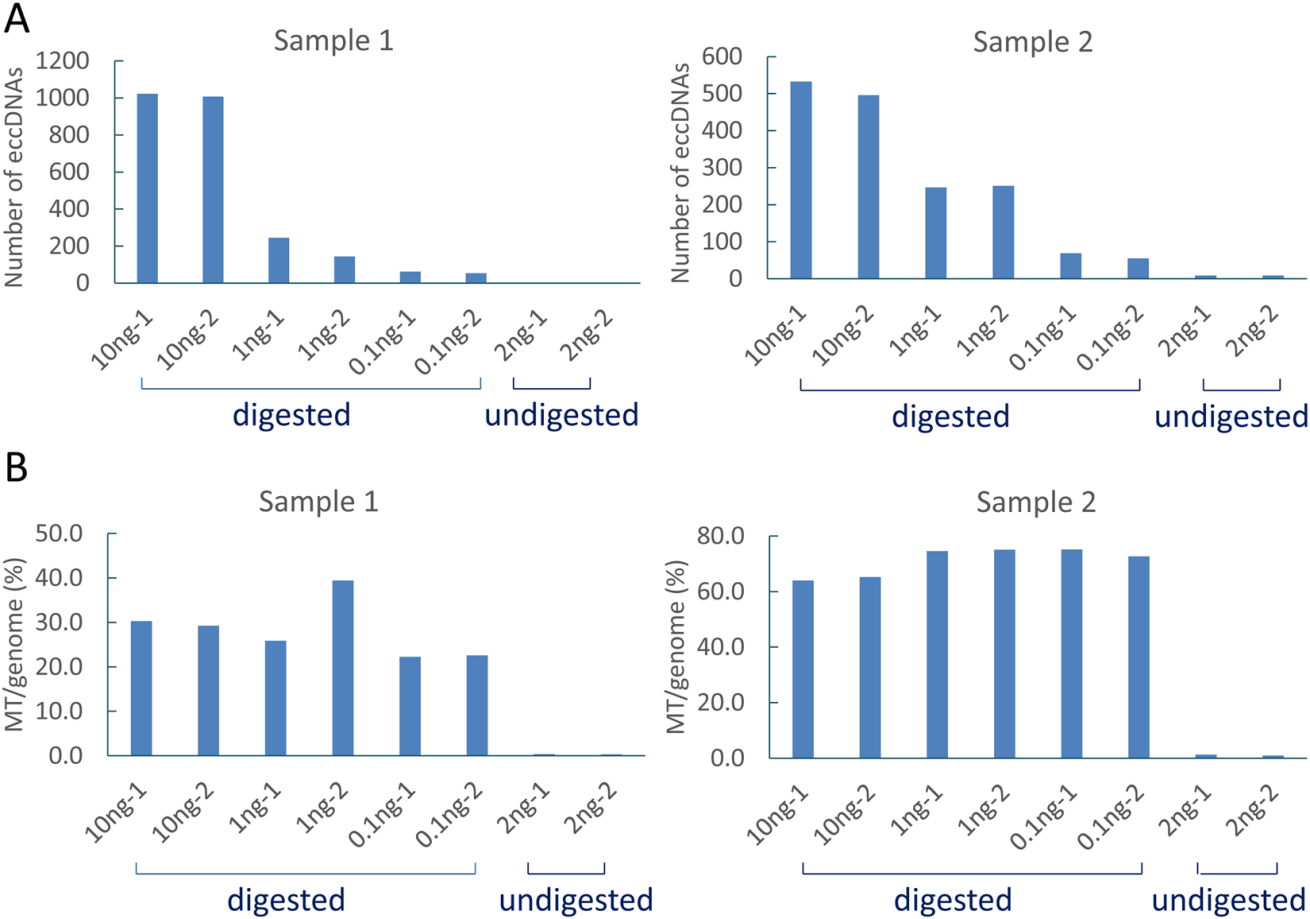
Effect of DNase digestion and initial DNA input on eccDNA detection. (**A**) Number of uniquely detected eccDNAs in different groups in samples 1 and 2. Compared to the digested groups, the undigested groups generated extremely low eccDNAs (3–9 for each assay). In the digested groups, eccDNA detection rate is reduced with the decreasing of initial cfDNA input. (**B**) Ratio between mapped mitochondria and genome sequence reads (MT/genome) in different groups in samples 1 and 2. The ratio of MT/genome decreased significantly in the undigested groups.

To test if ATP-dependent DNase treatment had any effect on eccDNA output, we compared the cfDNAs digested with ATP-dependent DNase to those without digestion. We found that the ATP-dependent DNase digestion significantly increased the detected eccDNAs. In samples 1 and 2, the DNase-digested group 3 (1 ng input) generated 144–251 (mean = 221) detectable eccDNAs while the non-digested group 4 (2 ng input) only generated 3–9 eccDNAs although both groups had similar numbers of human genome-mapped sequences (12~18 million of mapped sequence count) (Table 1, Fig. 3A). To evaluate effect of initial cfDNA input on eccDNA output, we divided cfDNAs into three groups with various inputs. We found significant reduction of detected eccDNAs when initial input decreased from 10 ng to 0.1 ng (Table 1, Fig. 3A). On average, we observed 8.3–17.4-fold more unique eccDNAs in 10 ng than in 0.1 ng input. We also compared technical replicates in all test conditions and observed similar numbers of detected eccDNAs (Table 1, Fig. 3A). However, few eccDNAs showed sequence overlaps between technical replicates, possibly due to the high heterogeneity of eccDNAs.

Meanwhile, as mitochondrial DNA stands for typical circular eccDNAs, we compared the read count ratios between mitochondria and human genome (MT/genome). We found significantly increased ratio of mitochondrial sequences after ATP-dependent DNase digestion. For samples 1 and 2, the ratio was 0.3–1.3% in non-digested groups and 22.3–75.1% in digested groups (Fig. 3B). Clearly, ATP-dependent DNase digestion is an important factor influencing eccDNA output. In sample 3, the MT/genome read count ratios in the digested groups were only 0.5–0.8%, which were similar to the undigested groups (0.8–0.9%) (Table 1). We believe that the low detection rate of eccDNAs and mitochondria DNA in sample 3 (5–15 eccDNA output in each assay) was caused by incomplete DNase digestion (Table 1, Fig. 3). Thus, we used the sample 1 and sample 2 in the following annotation analysis.

### Functional annotation of plasma eccDNAs

**Figure 4:**
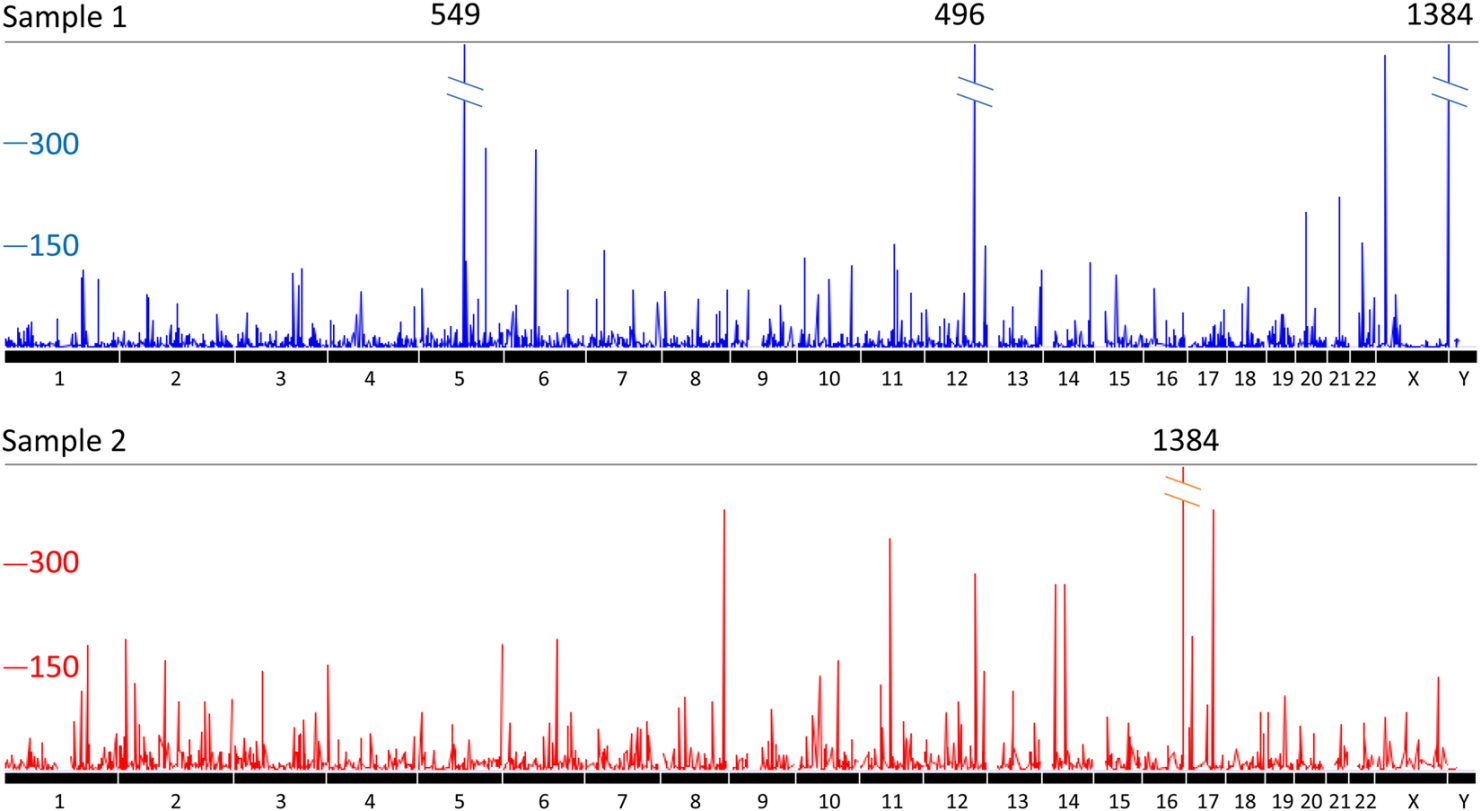
Genome view of eccDNA distribution in samples 1 and 2. The X axis stands for the 23 chromosomes. The Y axis shows the normalized read depth (normalized to total read count of all split reads with read depth ≥3).

To visualize distribution of all detected eccDNAs, we first transformed the eccDNA read counts based on all mapped split read counts. We then plotted the normalized read counts for each of the eccDNAs along human genome (Fig. 4). Although most eccDNA regions were not co-localized, we found five genomic regions that were overlapped between samples 1 and 2. These regions were located inside of four protein coding genes *COL4A3BP*, *NT5DC*1, *CREB5* and *CTXN2* and one RNA gene *CTB-12O2*.*1*.

**Figure 5:**
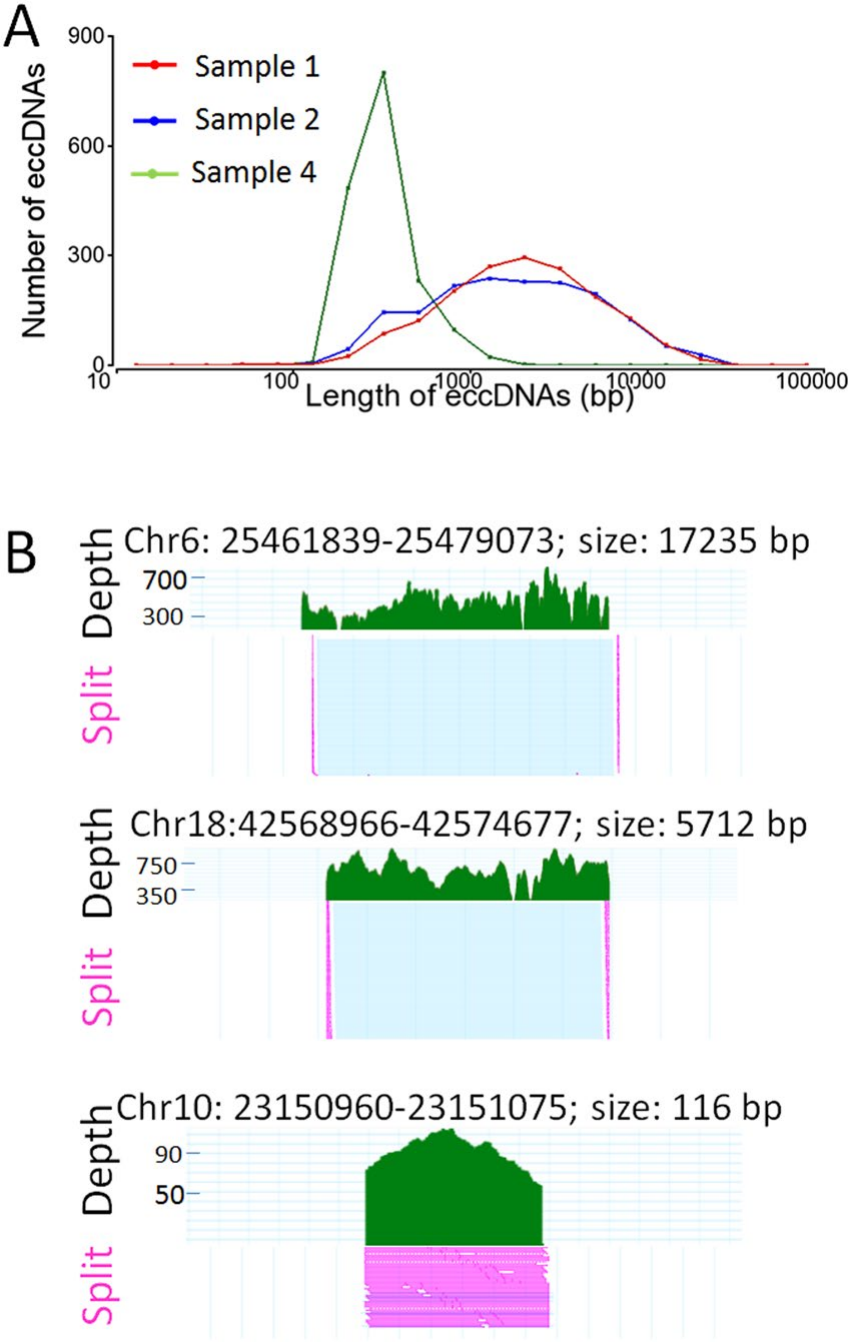
Size distribution and mapping characteristics of eccDNAs. (**A**) Size distribution of cell free eccDNAs in samples 1 (red line), 2 (blue line) and 4. For sample 1 and 2, the sizes of eccDNAs are from 31 bp to 19,989 bp (median 1801–1962 bp). The majority (≥80%) are smaller than 5000 bp. For sample 4, the majority (>89%) are less than 500 bp and the median size is 330 bp. (**B**) Representative mapped sequences in eccDNAs with large, medium and small sizes. From top to bottom, eccDNAs with 17,235 bp, 5,712 bp and 116 bp are shown along with the read depth and split reads (in pink).

To characterize the molecular features and predict the functional roles of these eccDNAs, we applied various bioinformatic tools and compared eccDNAs to a wide variety of genomic features. Unlike previous studies using cellular DNAs^3^, the current study did not perform size selection before MDA procedure. Therefore, we expect to observe the increased proportion of mitochondrial sequences. One advantage of this approach was to preserve the large size eccDNAs. We found that the eccDNA sizes were from 31 bp to 19,989 bp with the median size of 1801 and 1962 bp in samples 1 and 2, respectively. Most eccDNAs (≥80%) were smaller than 5000 bp (Fig. 5A). The large eccDNAs (>10 kb) accounted for 5.1% in sample 1 and 4.4% in sample 2. Among these eccDNAs in the two samples, the three longest eccDNAs with split read depth >10 were 18,192 bp (split reads = 15) at chr7: 19362586–19380777, 17,235 bp (split reads = 48) at chr6: 25461838–25479073, and 18,612 bp (split reads = 14) at chr22: 47621332–47639943. The representative eccDNAs with large, median and small sizes (17235 bp, 5712 bp and 116 bp) were shown in Fig. 5B.

**Figure 6:**
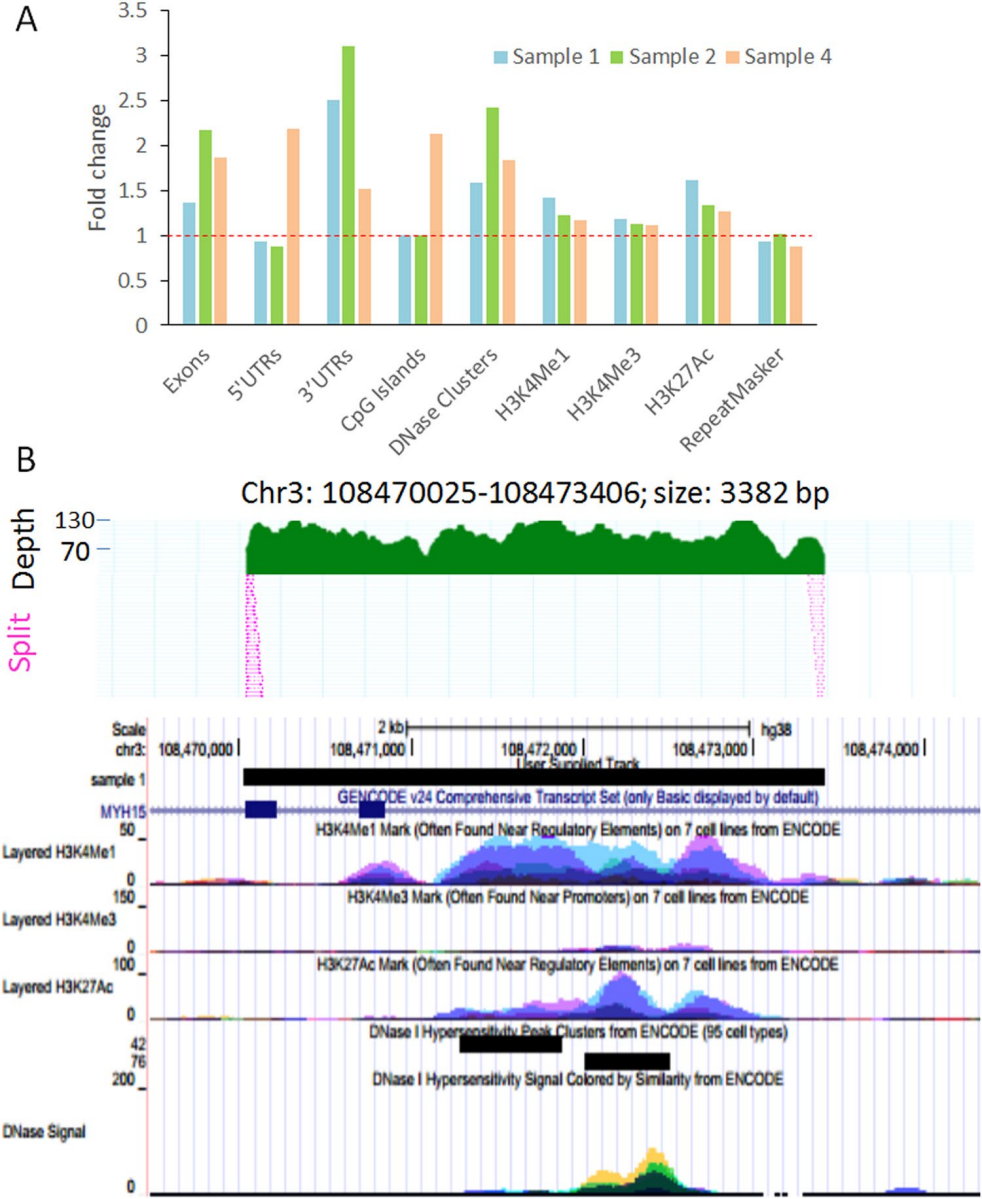
Functional annotation of eccDNA. (**A**) Genes (exons, 5′UTRs, 3′UTRs), regulatory elements (CpG Islands, DNase Clusters, H3K4Me1, H3K4Me3, H3K27Ac) and repetitive sequences (RepeatMasker) were intersected with eccDNAs (sized less than 1000 bp) in samples 1, 2 and 4. Random regions with matched chromosomes and lengths with eccDNAs were generated as random controls. The fold change stands for the ratio between elements counts in eccDNAs and the controls. Higher frequency of 3′UTRs, exons, DNase Clusters, H3K4Me1 and H3K27Ac are observed in eccDNA regions in all samples. Sample 4 also has higher enrichment of 5′UTRs and CpG island sequences. (**B**) Representative eccDNAs associated with genes and regulatory elements. Upper: Representative eccDNA with overall sequence coverage and split reads. Lower: Gene transcripts and epigenomic marks for regulatory DNA elements in the eccDNA region. The region covers two exons of *MYH15* gene. Histone marks of H3K4Me1, H3K4Me3, H3K27Ac, and DNase I hypersensitivity tracks from ENCODE are also shown.

Previous studies have shown that the eccDNAs may be originated from either repeat sequences or non-repeat sequences^3, 7, 8, 16–18^. To investigate the sequence components of newly identified circulating eccDNAs, we intersected the repetitive elements in Repeatmasker with eccDNAs as well as the random control genomic regions with chromosomes and sizes matched to eccDNAs. For both the total eccDNAs and those sized less than 1000 bp (710 and 556 eccDNAs in sample 1 and sample 2 respectively), we found equivalent numbers of repetitive elements in the eccDNA and the random control (0.928–1.006-fold change), indicating that the eccDNAs may not be generated extensively from repetitive elements. In contrast, we found enrichment of exons and 3′UTRs in eccDNAs with <1000 bp in size. When compared to random controls, fold enrichments were 1.36 and 2.5 for exons and 3′UTR in sample 1, and 2.17 and 3.1 in sample 2, suggesting that eccDNAs are more likely to originate from genic regions with active transcription (Fig. 6A). We also tested the intersection between regulatory elements (CpG Islands, DNase clusters, H3K4Me1, H3K4Me3, and H3K27Ac) and eccDNAs (<1000 bp) using UCSC Table Browser. By compared to random controls, we observed higher numbers of DNase Clusters, H3K4Me1, and H3K27Ac marks that are overlapped with the eccDNAs in both two samples. DNase Clusters inside of eccDNAs were increased by 1.58-fold (sample 1) and 2.42-fold (sample 2). H3K4Me1 and H3K27Ac enhancer marks showed 1.42–1.62-fold enrichments in eccDNAs of sample 1 and 1.23–1.33 fold enrichment of sample 2 (Fig. 6A). Example eccDNAs containing gene exons and regulatory elements are shown in Fig. 6B.

The overall mean GC content for all detected eccDNAs was ~42% for both samples 1 and 2. However, if the eccDNAs were divided into subgroups, GC content was significantly higher in smaller eccDNAs (size <500 bp, percentage in total eccDNAs = 11–17%) than larger ones (size >500 bp, percentage in total eccDNAs = 83–89%) (p < 0.01, t-test) (Supplementary Fig. S4). To predict the potential functional roles of eccDNAs, we applied DAVID annotation tool for the genes intersected with eccDNAs and observed significant enrichment of these genes in several categories including the Coiled coil, phosphoprotein, and fibronectin (p < 0.01), suggesting a potential involvement in gene expression regulation and cell adhesion events. GREAT (Genomic Regions Enrichment of Annotations Tool) analysis showed that the eccDNAs were more likely involved in genes associated with invasive cancer cell gene expression regulation (p < 0.001).

### Mechanisms of eccDNA formation

The current study identified a total of 2,542 and 1,669 junction sequences from the samples 1 and 2. To reveal the underlying mechanisms for junction formation, we selected top 500 sequences with highest split read depth to evaluate characteristics of the junction sequences for both samples. First, we examined NAHR (nonallelic homologous recombination) mechanism, which can generate rearrangements mediated by highly homologous repetitive sequences or segmental duplications (SDs, also called low-copy repeats, LCR))^19, 20^. Since the junction reads are split into two regions, we defined the breakpoint mapped to a smaller genomic coordinate as left breakpoint and the other as right breakpoint. By analyzing the 500 pairs of breakpoints in sample 1, we found that 206 left breakpoints and 216 right breakpoints were located inside of repeat elements (RepeatMasker at UCSC). Among these 422 repeat-containing breakpoints, there are 106 pairs with left and right breakpoints within the same eccDNAs. Among those, 14 pairs have the same type of repetitive elements (Supplementary Table 2). Additionally, for sample 1, we observed seven paired breakpoints located inside of the SDs. For sample 2, 16/500 pairs of breakpoints were located inside same type of repetitive elements and 6/500 pairs were inside of SDs. Thus, NAHR mechanism may play a role in circularization of broken DNA ends during eccDNA formation through repetitive elements or SDs. Representative graphs of these eccDNA breakpoints were shown in Supplementary Fig. S5.

We also evaluated nonhomologous end-joining (NHEJ) mechanism, a more prevalent pathway for error-prone repair of DNA double stand breaks (DSBs)^21^. NHEJ is characterized by the junctions of blunt-end, microhomology (may be defined as 1–3 bp) and small insertions (usually defined as <10 bp)^22–25^. For sample 1, by aligning top 500 junction sequences (after excluding those with paired breakpoints located inside same repeat sequences or SDs) to human genome and compared to each other at the broken ends, we observed 253 junctions with direct joining of the two ends (blunt ends rejoining) (50.6%), 195 junctions with microhomologies (1– bp) at the very end of the breakpoints (39%), and 5 junctions with small insertions of 1–7 bp (1%) (Supplementary Figs S6–S7). For sample 2, the percentage is 53.8% for blunt ends joining, 36.7% for 1–3 bp microhomology and 1.5% for small insertion (Supplementary Figs S6–S7). The data suggests that eccDNAs are frequently formed through simple blunt end joining or microhomologies, consistent with the NHEJ-mediated DNA repair. In addition, we observed 47 junction sequences in sample 1 and 39 junctions in sample 2 with microhomologies of 4–16 bp at the very end (8–0.4%) (Supplementary Figs S6 and S8). These sequences may indicate the mechanisms of microhomology-mediated end joining (MMEJ) or microhomology involved replication-based mechanisms.

### Further validation of circulating eccDNAs

To further validate our observation, we performed eccDNA analysis in an additional cfDNA sample (Sample 4) with greatly increased sequencing depth. eccDNAs in this sample were initially enriched with two technical replicates (Replicate 1 and Replicate 2) and then with two more replicates (Replicate 3 and Replicate 4). Each replicate used an input of 10ng cfDNA. For each replicate, we received 95 to 119 million raw read counts, and 88 to 109 million being mapped to the human genome, which is over 4-folds more mapped sequences than in samples 1 to 3. When using read depth >10 as a cutoff, unique eccDNA counts were 19508, 25723, 24764, 26069 in Replicates 1 to 4, respectively (Supplementary Table S3). The chromosome origins, the breakpoints on eccDNAs (left breakpoints, right breakpoints) and the estimated read depth, for all the four samples were listed in Supplementary Table S4.

To determine if these eccDNAs were overlapped in different replicates, we intersected the breakpoint regions (±20 bp of each breakpoint) of these eccDNAs between technical replicates and examined whether the intersected eccDNAs shared the same breakpoints. Among over 20 thousand of eccDNAs in each replicate, we observed only 12 eccDNAs with exactly same breakpoints between Replicates 1 and 2, and 15 eccDNAs between Replicates 3 and 4. Only two eccDNAs were shared in all four replicates. The detailed information of the same eccDNA captured by different replicates is listed in Supplementary Table S5. The genome view of eccDNAs in all four replicates is shown in Supplementary Fig. S9.

For Sample 4, the size of eccDNAs varied from ~30 bp to ~12 kb with median size of 330 bp (Fig. 5). Similar to samples 1 and 2, the GC content was higher in smaller eccDNAs (size <500 bp) than in larger eccDNAs (>500 bp) (p < 0.001, t-test). The intersection with genomic features including exons, 5′ and 3′ UTRs, CpG islands and DNase Clusters were higher (1.51–2.18 fold) when compared to random sequences with same length and chromosome origin (Fig. 6). The enrichment of histone markers (H3K4Me1, H3K4Me3, and H3K27Ac) were also slightly increased (1.11–1.27 fold) (Fig. 6). By contrast, the repetitive sequences in RepeatMaskers were not enriched in eccDNAs in Sample 4 compared to random controls (0.87 fold) (Fig. 6).

We also performed junction sequence analysis and found that 18 of 500 pairs of breakpoints (with top depth) were located inside same type of repetitive elements and 9 of 500 pairs were inside of SDs. Among 500 junctions locating on non-repetitive elements and SDs, 316 paired-ends were joined by blunt end rejoining (63.2%), 109 paired-ends were joined with 1–3 bp microhomology (21.8%), 38 paired-ends had longer microhomologies (>= 4 bp) (7.6%), and 37 ends showed small insertion (7.4%) (Supplementary Fig. S6).

## Discussion

eccDNAs are discovered in almost all karyocytes cells^4^. Previous studies characterize the eccDNAs using specific cell types including human cancer cell lines^7, 26^, animal^10, 27^ and yeast^2, 28^. These reported eccDNAs are all derived from inside cells. Extracellular DNAs (particularly, cfDNAs in blood) released by cell necrosis or apoptosis have emerged as a rich resource for biomarker discovery. However, non-mitochondrial eccDNAs in circulation system has not been reported. Due to relatively stable circular form, we tested our hypothesis that the cell free eccDNAs in blood system are prevalent and detectable. In this study, we applied sequencing technology and identified thousands of eccDNAs in blood plasma. We further described the distribution and functional characterization of eccDNAs. The wide spread presence in blood system provides evidence that the cell free eccDNAs may not only be functionally important but also serve as potential biomarkers for disease risk assessment, early detection and outcome prediction.

To enrich the circular DNAs from human plasma, we digested cfDNAs with ATP-dependent DNase and performed MDA to preferentially amplify undigested circular DNA. This strategy has been reported with high efficiency of capturing eccDNAs^2, 3, 29–31^. Due to low yield of cfDNAs in plasma, we skipped a step of mitochondrial DNA removal to reduce further DNA loss. This simplified method may be related with the broader size range of eccDNAs we enriched. In addition, the ratio between mitochondrial DNA and genome DNA (MT/genome) can be a sensitive measurement to determine the efficiency of circular DNA enrichment. In our experiments, the ATP-dependent DNase-digested groups show 80–130-fold higher ratio (MT/genome) when compared to the non-digested groups, suggesting highly efficient enrichments of circular DNAs. Instead of removing mitochondrial DNAs before library preparation, the simplified method can completely exclude mitochondrial DNAs by sequence mapping. The data presented in this study demonstrate that the strategy is feasible with high efficiency.

Our results showed that cfDNA input and ATP-dependent DNase digestion have significant effect on the detection of eccDNAs. The unique eccDNAs detected increased as the increasing of cfDNA input (two samples, 12 assays). The undigested groups generated much less eccDNAs and mitochondria DNA (two samples, four assays) than the digested groups (two samples, six assays). Low eccDNA output from sample 3 can be best explained by the bad digestion because the MT/genome ratios are very low in all the six assays in the digested groups, just like the case in the undigested groups, for this sample. Thus, higher cfDNA input and complete digestion are the key to capture eccDNAs effectively.

In our initial two samples (samples 1 and 2), we observed few overlaps of detected eccDNAs not only among samples but also between technical replicates. To confirm this observation, we added an additional sample and significantly increased the sequencing depth. Although the increased sequencing depth resulted in a few shared eccDNAs between replicates, the total number of such shared eccDNAs is still extremely low. Genomic bin method was used by some study to identify overlapped eccDNAs in technical replicates- sequences located inside the same genomic window being considered as overlapped eccDNAs^32^. Besides, research in eccDNAs in yeast compared the genes on eccDNAs that are common in different samples and identified 12–16% of eccDNA-related genes overlapped among biological samples^2^. Although these studies showed higher overlap sequences, none of them showed breakpoints and junction sequences at base pair level to determine if two eccDNAs have the same sequences. Therefore, our study is the first to compare the exactly same eccDNAs between technical replicates. Our results strongly support the presence of a huge number of heterogeneously-structured eccDNAs in human cfDNAs.

Many eccDNAs are generated extensively from repeat sequences like the tel-eccDNA^8, 16–18^. Some eccDNAs can be unique molecules with functional or non-random sequences. For example, the large circular formed DNA-DMs are usually the vehicles of amplification of oncogene and drug resistance genes; while the small sized microDNA are composed of sequences prone to be genic regions. Like microDNAs, the cell free eccDNA are also enriched in UTRs, and exon regions. Additionally, enrichments at DNase Clusters and H3K4Me1 and H3K27Ac marks suggest potential regulatory roles of these eccDNAs in RNA transcription or protein translation. By providing the binding sites for the transcription factors and chromatin-modifying complexes, the eccDNAs may serve as a potential expression regulator or a template for promoterless transcription^33^. Specific forms of eccDNAs such as the DMs, episomes and spcDNA have been proved to be associated with elevated gene expression and enhanced chromosome instability^34, 35^. Additionally, tissue specificity of eccDNAs has been suggested by comparing different organ sites (ovarian, prostate)^7^. Our data further support potential involvement of eccDNAs in cell adhesion events and cancer gene expression process. The possible role of eccDNAs participating in regulatory network to tune cell function need to be further addressed.

Junction sequences after DNA circularization are important for underlying mechanisms of eccDNA formation^24^. So far, knowledge on mechanisms of eccDNA generation is limited. Proposed repair mechanisms include both the replication-based mechanisms and non-replicative mechanisms^36–40^. The non-replicative repair mechanisms mainly include NAHR, NHEJ and MMEJ. NAHR relies on SDs to generate recurrent rearrangements and the highly homologous repetitive sequences (Alu, L1) to generate the non-recurrent rearrangements^19, 20^. NHEJ (or classical NHEJ, c-NHEJ) has the junctions characterized with blunt end joining, microhomology (1–3 bp) and small insertions while MMEJ is based on the microhomology sequences at the break ends. Definition for length of microhomology in MMEJ varies from 1 to 25 bp (more frequently 5–9 bp)^22, 41^. Replication-based mechanisms include the replication slippage and FosTes/MMBIR mechanisms. FosTes/MMBIR mechanisms are more frequently associated with complicated recombination. All these mechanisms require short microhomology sequences (defined as 2–5 bp for FosTes/MMBIR)^42–45^. For the eccDNAs extensively overlapping with the repetitive sequences, homologous recombination (HR) mechanism has been proposed^8, 9^. But other mechanisms such as NHEJ, MMEJ, and replication slippage have also been proposed^36–38^. In many cases, heterogenous mechanisms are involved for eccDNA formation^2, 7^. In this study, we show that a small fraction of the paired breakpoints are located inside of the same repeats (2.8–3.6%) and SDs (1.2–1.8%), which may facilitate the NAHR mechanism. In contrast, majority of the junctions are in favor of NHEJ pathway with blunt end joining, 1–3 bp microhomology or small insertions. The microhomology sequences, especially those with length of 4–16 bp microhomology (38–47/500 from this study), suggest that MMEJ, replication slippage may also be involved in the genesis of eccDNAs.

In summary, we applied a novel sequencing technology to determine cell-free eccDNAs in plasma samples. Using split reads as discovery tool, we identified tens of thousands of eccDNAs with a wide range of sizes and abundances. Plasma eccDNAs are highly heterogenous but not generated randomly. Instead, eccDNAs are prone to be generated from genic and regulatory regions. The eccDNAs are relatively stable in circulation system and may be associated with genome instability in tumor and other diseases. Quantification of certain eccDNAs may reflect disease status and can be used as biomarkers for disease assessment. This is the first study to systematically evaluate cell-free eccDNAs in circulation system. The potential application of eccDNAs as new extracellular nucleic acid biomarkers for cancer and other diseases deserves further exploration.

## Methods

### Study design

Overall study design is shown in Fig. 1. To enrich eccDNAs, we treated cfDNA with ATP-dependent DNase digestion to remove linear genomic DNAs and performed a Multiple Displacement Amplification (MDA) method to amplify undigested circular DNAs. We used the amplified DNAs to prepare sequencing libraries^3, 7, 31^. To estimate technical variations and specificity of the methods, we extracted plasma cfDNAs from three blood donors and included technical replicate in each assay. For each sample, the cfDNA was digested using ATP-dependent DNase. After that, the digested cfDNA was divided into groups of 10ng (Group 1), 1ng (Group 2) and 0.1ng (Group 3), calculated from the initial amount of cfDNA. Group 4 used 2 ng cfDNA without ATP-dependent DNase digestion. Additionally, a negative control group was included (H_2_O from the REPLI-g Single Cell Kit). The digested, undigested cfDNA as well as negative control were amplified with the MDA method. The MDA products were purified, sonicated and used for library construction. Together, we prepared 26 sequencing libraries (13 replicates) from three cfDNA samples (samples 1, 2, 3) and the control group (H_2_O). Besides we made four libraries (Replicates 1, 2, 3, and 4) with input of 10ng cfDNA for sample 4.

### Plasma sample collection and preparation

Peripheral blood samples from three donors were collected in 10 ml plasma separator tubes. Within 2 hours after collection, the plasma samples were fractioned into multiple aliquots after centrifugation at 2,000 × g for 10 min, leaving >1 ml supernatant and cell debris intact in the original tubes. All plasma samples were stored at − 80 °C until use. Informed consent was obtained from the study participants prior to blood draw. This study was approved by Institution Review Board at Medical College of Wisconsin. All experiments were performed in accordance with relevant guidelines and regulations.

### eccDNA enrichment and amplification

We extracted cfDNA from plasma samples using QIAamp DNA Blood Maxi Kit (Qiagen, 51192). The cfDNA was subjected to Plasmid-Safe^TM^ ATP-dependent DNase (Epicentre, E310K) digestion at 37 °C for 1.5 hours with a final concentration of 0.4 U/µl to remove linear dsDNA. After inactivating the ATP-dependent DNase at 70 °C for 30 min, we amplified the remaining cfDNA using the REPLI-g Single Cell Kit (Qiagen, Cat #150343) at 30 °C for 8 hours. The amplified products were purified using Agencourt AMPure XP (Beckman Coulter, A63881) and quantified using iQuant™ High Sensitivity dsDNA Quantitation Kit (GeneCopoeia, N011).

### Sequencing library preparation and data analysis

eccDNA-enriched DNAs were first sheared into 100–300 bp by sonication (Covaris, Inc. Woburn, MA). 30ng of the sheared DNA was subjected to DNA library preparation using the ThruPLEX® DNA-seq kit (Rubicon Genomics, Ann Arbor, MI). for samples 1, 2 and 3, 26 indexed libraries were sequenced in one lane with 125 bp paired-end sequencing (Illumina HiSeq. 2500). For sample 4, four libraries were sequenced in one lane. The sequencing results were analyzed using DNASTAR (Madison, WI). As mitochondrial DNA was also enriched, any sequences that were aligned to the mitochondrial DNA sequences were removed before mapping to human genome (hg38). A custom-created program, named “split-align”, was used to identify sequences with split reads, defined as a complete sequence read that is split into two sub-sequences, each being at least 25nt long and mapping to a different genomic region in the same strand of a chromosome. Different from genomic deletions, the two sub-sequences for eccDNAs are inconsistently mapped, with first sub-sequence read direction toward away from second sub-sequence. For sample 1, 2 and 3, the split reads with sequence depth ≥3 were used as a cutoff; for sample 4, split read depth >10 were used as a cutoff. The procedure for eccDNA enrichment, library preparation, DNA sequencing and data analysis is illustrated in Fig. 2.

### Split junction sequence verification

To verify the junction sequences of eccDNAs identified, nine pairs of PCR primers specific for breakpoint junctions were designed using Primer 3 (http://bioinfo.ut.ee/primer3–0.4.0/primer3/). PCR experiments were performed using Taq DNA Polymerase with Standard Taq Buffer (NEB, M0273). For a 15 µl reaction, the PCR reaction system included 10 ng of the MDA products with initial denaturing step of 95 °C for 2 min; 35 cycles of 95 °C for 30 s, 55–65 °C for 30 s, 68 °C for 30 s; and a final extension of 68 °C for 5 min. The PCR products were analyzed by agarose gel electrophoresis. Primer sequences, expected PCR product size and raw sequencing depth of the split reads are shown in Supplementary Table 1.

### Enrichment analysis and functional annotation of eccDNAs

To reveal the prevalence of repetitive sequences, repetitive elements in RepeatMasker integrated in UCSC (hg 38) were intersected with eccDNAs and the random controls. The random controls were generated using the random regions with the chromosome and length matched to the eccDNAs. To determine if eccDNAs were enriched in specific genomic regions, table browser from UCSC was used to download sequence coordinates for 5′UTRs, 3′UTRs and exons (hg 38).

eccDNAs and the random controls were intersected with the UTRs, exons and epigenomic elements, including CpG islands, DNase Clusters, H3K4Me1 (GM12878), H3K4Me3 (K562) and H3K27Ac (K562) marks from UCSC ENCODE Regulation Super-track (hg 38). Fold enrichment at these sites were calculated compared to the random controls. Genomic region enrichments were analyzed using bedtools from Galaxy (https://usegalaxy.org/) and GREAT (Genomic Regions Enrichment of Annotations Tool). DAVID (the Database for Annotation, Visualization and Integrated Discovery) was used for enrichment analysis of eccDNAs-related genes. The junction sequences were aligned using UCSC blat tool and manually annotated for their junction types (blunt end/microhomology/insertion).

### Data Availability

The raw sequence data of all samples were uploaded to Sequence Read Archive (SRA) database (https://www.ncbi.nlm.nih.gov/sra) (SRP115110). The datasets generated and analyzed during the current study are available from the corresponding author on reasonable request.

## Electronic supplementary material

Supplementary Information

Supplementary Table S4

## References

[1] Kuttler F, Mai S (2007). Formation of non-random extrachromosomal elements during development, differentiation and oncogenesis. Semin Cancer Biol.

[2] Moller HD, Parsons L, Jorgensen TS, Botstein D, Regenberg B (2015). Extrachromosomal circular DNA is common in yeast. Proc Natl Acad Sci USA.

[3] Shibata Y (2012). Extrachromosomal microDNAs and chromosomal microdeletions in normal tissues. Science.

[4] Cohen S, Segal D (2009). Extrachromosomal circular DNA in eukaryotes: possible involvement in the plasticity of tandem repeats. Cytogenet Genome Res.

[5] Gaubatz JW (1990). Extrachromosomal circular DNAs and genomic sequence plasticity in eukaryotic cells. Mutat Res.

[6] Zhu J (2013). De novo-generated small palindromes are characteristic of amplicon boundary junction of double minutes. Int J Cancer.

[7] Dillon LW (2015). Production of Extrachromosomal MicroDNAs Is Linked to Mismatch Repair Pathways and Transcriptional Activity. Cell Rep.

[8] Cohen S, Yacobi K, Segal D (2003). Extrachromosomal circular DNA of tandemly repeated genomic sequences in Drosophila. Genome Res.

[9] Cohen S, Houben A, Segal D (2008). Extrachromosomal circular DNA derived from tandemly repeated genomic sequences in plants. Plant J.

[10] Cohen Z, Bacharach E, Lavi S (2006). Mouse major satellite DNA is prone to eccDNA formation via DNA Ligase IV-dependent pathway. Oncogene.

[11] Mandel PPM (1948). Les acides nucleiques du plasma sanguin chez l’homme. C R Seances Soc Biol Fil.

[12] Jung K, Fleischhacker M, Rabien A (2010). Cell-free DNA in the blood as a solid tumor biomarker–a critical appraisal of the literature. Clin Chim Acta.

[13] Fleischhacker M, Schmidt B (2007). Circulating nucleic acids (CNAs) and cancer–a survey. Biochim Biophys Acta.

[14] Schwarzenbach H, Hoon DS, Pantel K (2011). Cell-free nucleic acids as biomarkers in cancer patients. Nat Rev Cancer.

[15] Diaz LA, Bardelli A (2014). Liquid biopsies: genotyping circulating tumor DNA. J Clin Oncol.

[16] Cohen S, Agmon N, Sobol O, Segal D (2010). Extrachromosomal circles of satellite repeats and 5S ribosomal DNA in human cells. Mob DNA.

[17] Navratilova A, Koblizkova A, Macas J (2008). Survey of extrachromosomal circular DNA derived from plant satellite repeats. BMC Plant Biol.

[18] Cohen S, Mechali M (2002). Formation of extrachromosomal circles from telomeric DNA in Xenopus laevis. EMBO Rep.

[19] Cardoso AR, Oliveira M, Amorim A, Azevedo L (2016). Major influence of repetitive elements on disease-associated copy number variants (CNVs). Hum Genomics.

[20] Gu W, Zhang F, Lupski JR (2008). Mechanisms for human genomic rearrangements. Pathogenetics.

[21] Kasparek TR, Humphrey TC (2011). DNA double-strand break repair pathways, chromosomal rearrangements and cancer. Semin Cell Dev Biol.

[22] McVey M, Lee SE (2008). MMEJ repair of double-strand breaks (director’s cut): deleted sequences and alternative endings. Trends Genet.

[23] Shibata A (2009). Role of Parp-1 in suppressing spontaneous deletion mutation in the liver and brain of mice at adolescence and advanced age. Mutat Res.

[24] Carvalho CM, Lupski JR (2016). Mechanisms underlying structural variant formation in genomic disorders. Nat Rev Genet.

[25] Pannunzio NR, Li S, Watanabe G, Lieber MR (2014). Non-homologous end joining often uses microhomology: implications for alternative end joining. DNA Repair (Amst).

[26] Meng X (2015). Novel role for non-homologous end joining in the formation of double minutes in methotrexate-resistant colon cancer cells. J Med Genet.

[27] Zou H (2015). Double minute amplification of mutant PDGF receptor alpha in a mouse glioma model. Sci Rep.

[28] Moller HD (2015). Formation of Extrachromosomal Circular DNA from Long Terminal Repeats of Retrotransposons in Saccharomyces cerevisiae. G3 (Bethesda).

[29] Yamagishi H (1983). Purification of small polydisperse circular DNA of eukaryotic cells by use of ATP-dependent deoxyribonuclease. Gene.

[30] Lanciano S (2017). Sequencing the extrachromosomal circular mobilome reveals retrotransposon activity in plants. PLoS Genet.

[31] Moller, H. D. *et al*. Genome-wide Purification of Extrachromosomal Circular DNA from Eukaryotic Cells. *J Vis Exp* (2016). 10.3791/54239 PMC4841354 27077531

[32] Massa Shoura, I. G., Loren, H., Jason, M., Jason, G. & Stephen, L. Andrew Fire. Beyond The Linear Genome: Comprehensive Determination Of The Endogenous Circular Elements In C. elegans And Human Genomes Via An Unbiased Genomic-Biophysical Method. *bioRxiv* (2017).

[33] Seidl CI, Lama L, Ryan K (2013). Circularized synthetic oligodeoxynucleotides serve as promoterless RNA polymerase III templates for small RNA generation in human cells. Nucleic Acids Res.

[34] Storlazzi CT (2006). MYC-containing double minutes in hematologic malignancies: evidence in favor of the episome model and exclusion of MYC as the target gene. Hum Mol Genet.

[35] Cohen S, Regev A, Lavi S (1997). Small polydispersed circular DNA (spcDNA) in human cells: association with genomic instability. Oncogene.

[36] van Loon N, Miller D, Murnane JP (1994). Formation of extrachromosomal circular DNA in HeLa cells by nonhomologous recombination. Nucleic Acids Res.

[37] Galeote V (2011). Amplification of a Zygosaccharomyces bailii DNA segment in wine yeast genomes by extrachromosomal circular DNA formation. PLoS One.

[38] Vogt N (2004). Molecular structure of double-minute chromosomes bearing amplified copies of the epidermal growth factor receptor gene in gliomas. Proc Natl Acad Sci USA.

[39] Cohen S, Mechali M (2001). A novel cell-free system reveals a mechanism of circular DNA formation from tandem repeats. Nucleic Acids Res.

[40] Cohen Z, Lavi S (2009). Replication independent formation of extrachromosomal circular DNA in mammalian cell-free system. PLoS One.

[41] Sfeir A, Symington LS (2015). Microhomology-Mediated End Joining: A Back-up Survival Mechanism or Dedicated Pathway?. Trends Biochem Sci.

[42] Viguera E, Canceill D, Ehrlich SD (2001). Replication slippage involves DNA polymerase pausing and dissociation. EMBO J.

[43] Hastings PJ, Ira G, Lupski JR (2009). A microhomology-mediated break-induced replication model for the origin of human copy number variation. PLoS Genet.

[44] Lee JA, Carvalho CM, Lupski JRA (2007). DNA replication mechanism for generating nonrecurrent rearrangements associated with genomic disorders. Cell.

[45] Zhang F (2009). The DNA replication FoSTeS/MMBIR mechanism can generate genomic, genic and exonic complex rearrangements in humans. Nat Genet.

