# Supplementary material for: Molecular characterization of cell-free eccDNAs in human plasma
Source: Sci Rep. 2017 Sep 8;7:10968. doi: 10.1038/s41598-017-11368-w (PMC5591271; doi:10.1038/s41598-017-11368-w)
Supplement: Supplementary file 1 — Supplementary Information [file 41598_2017_11368_MOESM1_ESM.pdf]

Molecular characterization of cell-free eccDNAs in human plasma

Jing Zhu[1,2], Fan Zhang[3], Meijun Du[2], Peng Zhang[2], Songbin Fu[1], Liang Wang[2*]

[1]Laboratory of Medical Genetics, Harbin Medical University, Harbin, Heilongjiang, China. 150081.
[2]Department of Pathology and MCW Cancer Center, Medical College of Wisconsin, Milwaukee, WI 53226, USA
[3]School of Computer Science and Technology, Harbin Institute of Technology, Harbin, Heilongjiang, China.150001

*correspondence:
Liang Wang, MD, PhD.
TBRC-C4970
8701 Watertown Plank Road
Medical College of Wisconsin
Milwaukee, WI 53226
Phone: (414) 955-2574
Email: liwang@mcw.edu

Supplementary Table S1. Primer and product information for junction sequence verification.

	Forward primer	Reverse primer	Product size (bp)	Estimated Depth
J1	CGCTATTATTGCCATTATGTATTTG	TTTGAGGGTATTTTGTACGTTAGAAG	91	3
J2	AGTGTGGCTGGAAAGTGACT	CATGCTTGACTACTGGCTGC	93	102
J3	TCTCGCCGCACTGTTATTTC	AGTATGCCTGATTCATTTCCAGA	100	57
J4	TACCAGAGTTCCAACCCACC	GTCCATGTTATTTCCCTGGCA	112	549
J5	TCTGCTACTAAGTGTGACTCAGTGAT	CAGTAAATTTTTGTGAGGTTCTGC	99	10
J6	AGGAAGGTGACAGAGGGAGA	CTGTTGGCCATTTCCTGAGT	93	3
J7	ACACACCTGTAATCCCAGC	CAAGAAGAGGCTCCAGGAGA	95	152
J8	CGGCTGAGGTTACCCAAGG	GTGTCTGCCTCACCTCCTTA	101	109
J9	CTGGGTGCTGCATGGGTC	CCAAATTCCTGCTCCCACC	100	1384

Supplementary Table S2. eccDNAs with both breakpoints located inside of same type of repetitive elements in sample 1.

Chromosome	Left breakpoint	Right breakpoint	Repeat name	Repeat family	Repeat class
chr1	36454214	36455477	L2	L2	LINE
chr1	147395307	147397169	L1MD1	L1	LINE
chr2	123444866	123444894	LTR49	ERV1	LTR
chr2	132967634	132971585	L2	L2	LINE
chr3	104927021	104927406	LTR16A	ERVL	LTR
chr5	46562588	46562949	ALR/Alpha	centr	Satellite
chr5	77454199	77454375	MLT1F1	ERVL-MaLR	LTR
chr8	1604530	1604572	MER5A	hAT-Charlie	DNA
chr8	83414053	83414993	L1MC3	L1	LINE
chr12	127584013	127584705	L1M4	L1	LINE
chrX	18798703	18798968	AluSg	Alu	SINE
chrX	44860885	44861759	L1M6	L1	LINE
chrX	40527380	40528616	ERV3-16A3_I-int	ERVL	LTR
chrY	19761877	19762899	L1PA15	L1	LINE

Supplementary Table S3. Statistical summary of eccDNA sequencing data in Sample 4.

Sample name	Replicate name	Raw read count	Mapped sequence count	Mapped genomic (MT-removed)	Mapped MT DNA	Split read counts	Number of eccDNA	MT/genome (%)
Sample 4	Replicate 1	95,636,188	88,237,946	65,024,837	23,213,109	3,660,541	19,508	26.3
	Replicate 2	108,052,748	99,526,315	79,247,082	20,279,233	4,489,572	25,723	20.4
	Replicate 3	119,650,726	109,511,675	93,472,193	16,039,482	5,022,349	24,764	14.6
	Replicate 4	119,040,182	108,552,757	94,500,655	14,052,102	4,748,009	26,069	12.9

Supplementary Table S4. Chromosome origins, breakpoints and read depth of eccDNAs for samples 1, 2, 3 and 4. (See Excel table of Supplementary Table S4).

Supplementary Table S5. Overlapped eccDNAs in replicative assays in Sample 4.

Chromosome	Left breakpoint	Right breakpoint	Captured in replicates
chr1	23635248	23635449	R1 and R2
chr1	244236203	244236532	R1 and R2
chr5	1905508	1905693	R1 and R2
chr5	141292947	141293282	R1 and R2
chr6	122347864	122348057	R1 and R2
chr6	134828724	134828926	R1 and R2
chr6	160137773	160137962	R1 and R2
chr7	100671052	100671427	R1 and R2
chr7	149862001	149862319	R1 and R2
chrX	88300868	88301046	R1 and R2
chr21	8242643	8470543	R1 and R2
chr21	42313749	42313951	R1 and R2
chr2	27017449	27017764	R3 and R4
chr2	118385401	118385743	R3 and R4
chr2	134405916	134406242	R3 and R4
chr2	157989386	157989579	R3 and R4
chr3	42033150	42033480	R3 and R4
chr8	37626083	37626426	R3 and R4
chr8	53460110	53460300	R3 and R4
chr9	130315694	130315897	R3 and R4
chr11	64567184	64567390	R3 and R4

chr11	64567189	64567393	R3 and R4
chr13	99516115	99516459	R3 and R4
chr17	43903742	43903925	R3 and R4
chr20	46839374	46839707	R3 and R4
chr1	154750235	154750422	R1, R2, R3 and R4
chr6	39078387	39078576	R1, R2, R3 and R4

Supplementary Figure S1.

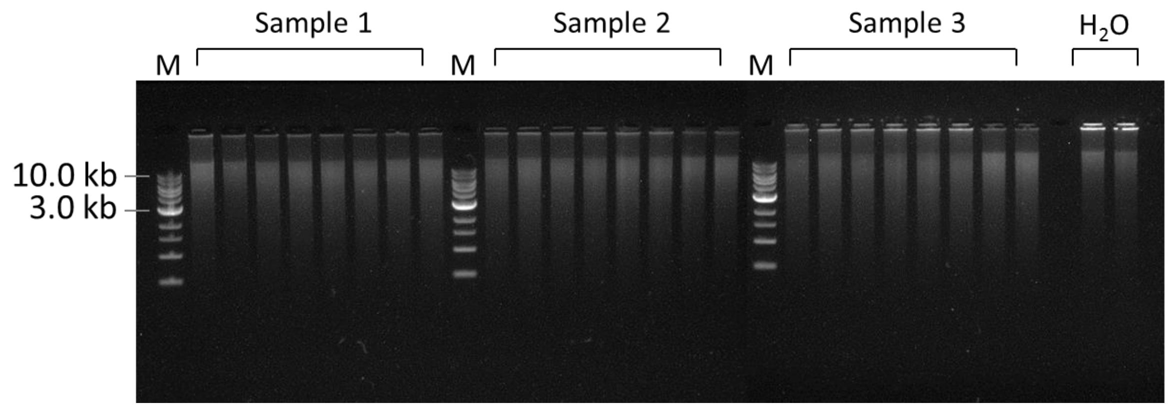

Supplementary Figure S1. Agarose gel electrophoresis of MDA products. The MDA products were first purified using AMPure XP beads and then analyzed using the 0.8% agarose gel electrophoresis. The results showed that each assay including H_2O had the products sized above 10 kb. The non-specific amplification from H_2O was confirmed by sequencing the amplified products. M: 1 kb DNA ladder (NEB).

Supplementary Figure S2.

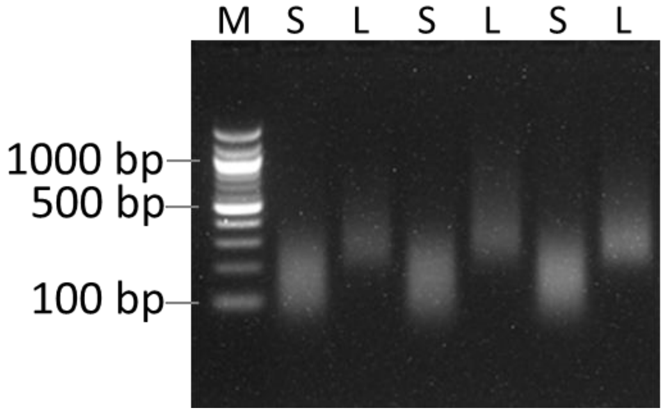

Supplementary Figure S2. Agarose gel electrophoresis of the sonicated MDA products and DNA libraries. The MDA products were sheared into100-300 bp in size (lane S). The DNA libraries from sheared DNA fragments were 250-500 bp (lane L). S: sonicated DNA fragments. L: DNA libraries. M: 100 bp DNA ladder (NEB).

Supplementary Figure S3.

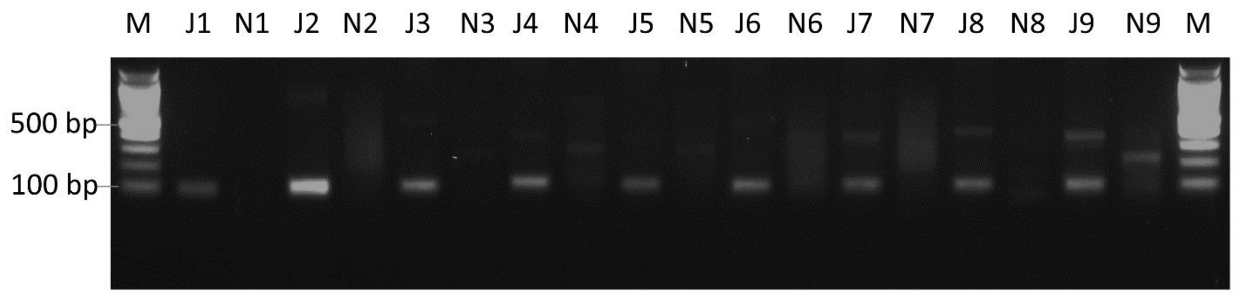

Supplementary Figure S3. Verification of eccDNA junctions using PCR and gel electrophoresis. Junction-specific PCRs were performed using MDA-derived products and normal human DNA as template DNA. The PCR products from eccDNAs (J1 to J9 lanes) and from normal human DNA (N1 to N9 lanes) were analyzed in 1.8% agarose gel. Positive bands with expected sizes were seen in the J1 to J9 lanes but not in the N1 to N9 lanes.

Supplementary Figure S4.

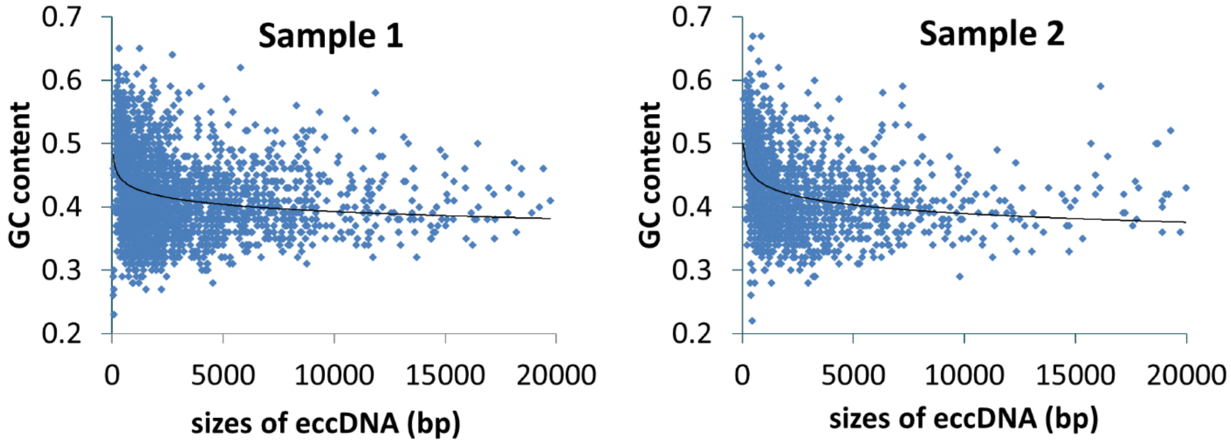

Supplementary Figure S4. GC content and eccDNA sizes. The smaller eccDNAs (<500 bp) show a higher GC content than the larger ones (>500 bp) in both samples 1 and 2 ($p < 0.01$, t-test).

Supplementary Figure S5.

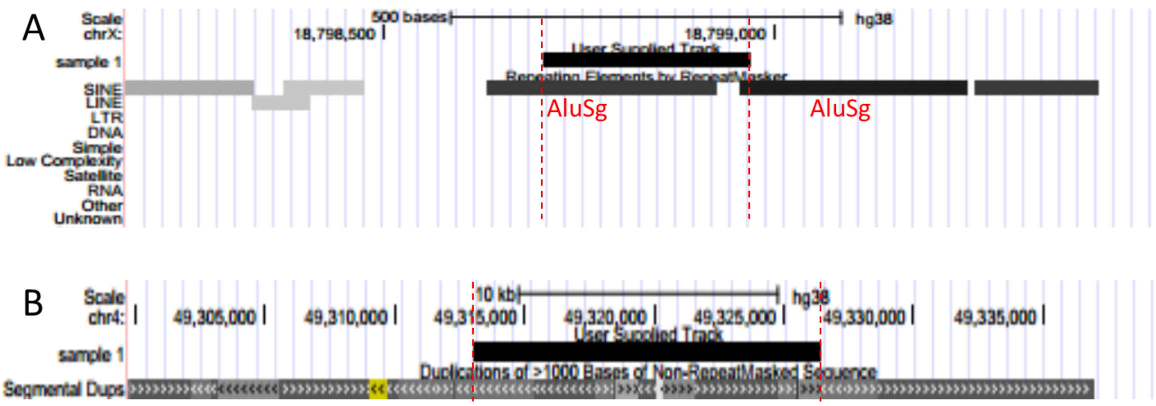

Supplementary Figure S5. Representative eccDNAs with both ends mapped to the same type of repetitive elements or segmental duplications. Breakpoints were intersected with repetitive elements or the segmental duplications using RepeatMasker in UCSC genome browser. Breakpoints located inside of the same type of repetitive elements are shown in A and located inside of segmental duplications shown in B. Red dashed lines across the two ends indicate the breakpoint regions. The names of repetitive elements are labeled just below the element bars.

Supplementary Figure S6.

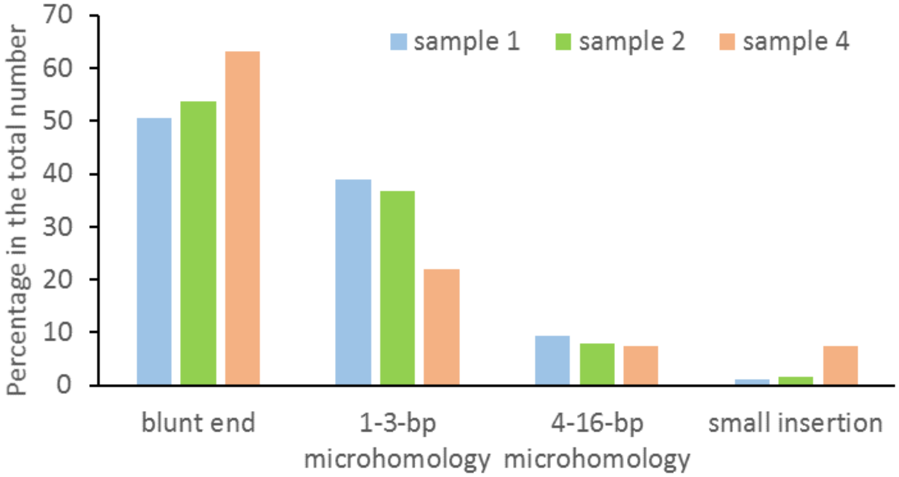

Supplementary Figure S6. Characteristics of junction sequences in samples 1, 2 and 4. The junction sequences were aligned to the human genome and compared for sequence characteristics. The junction types are similar in samples 1,2 and 4. Blunt end joining accounts for 50.6-63.1% of the junction sequences, 1-3 bp microhomology for 21.9-39%, 4-16 bp microhomology for 7.5-9.4%, and 1-7 bp insertions for 1-7.5%.

Supplementary Figure S7.

```
           Chr8 (-) 139981547                                  Chr8 (-) 139981500
Reference  AACCGTGTGGCGCCCAGTCTTCTTGCAGGTTGTTATTGAGGCTTATTT
Junction A AACCGTGTGGCGCCCAGTCTTCTTTGTCTCTCCTTAGTGAAACCGCAT
Reference  CCATGCACAACCGGAAACAGTGGATGTCTCTCCTTAGTGAAACCGCAT
           Chr8 (-) 139982229                                  Chr8 (-) 139982182

           Chr10 (+) 93618217                                  Chr10 (+) 93618264
Reference  TTCAGGAAAGCCTAGAAAAGTACTAAGTTTCTCTTCCTAGTACTTACT
Junction B TTCAGGAAAGCCTAGAAAAGTACCATATCCCTGACCACACACAACTTT
Reference  CTTGGAGGCCTCCCTCTTTCTACCATATCCCTGACCACACACAACTTT
           Chr10 (+) 93616212                                  Chr10 (+) 93616259

           Chr4 (-) 4334722                                    Chr4 (-) 4334674
Reference  TTCTGAGGAATCTCCGAACCATTTTCTGCAGTGGCTGCAGCACTTTCCA
Junction C TTCTGAGGAATCTCCGAACCATTATAGAGGGCTCCCTGGTCTCGCGGTA
Reference  TCTGTCACGCCCAGACAGGGCCACTAGAGGGCTCCCTGGTCTCGCGGTA
           Chr4 (-) 4335458                                    Chr4 (-) 4335410
```

Supplementary Figure S7. Representative junction sequences in favor of NHEJ mechanism. Junctions A, B and C show the types of blunt end joining, 1-3 bp microhomology and small insertion, respectively. Reference stands for the normal human reference sequence. Positions of the start and end of the reference sequence are shown on the above (green) and below (red) of sequences. Bases highlighted in yellow are the micro-homology sequences. Base in pink is the small insertion.

Supplementary Figure S8.

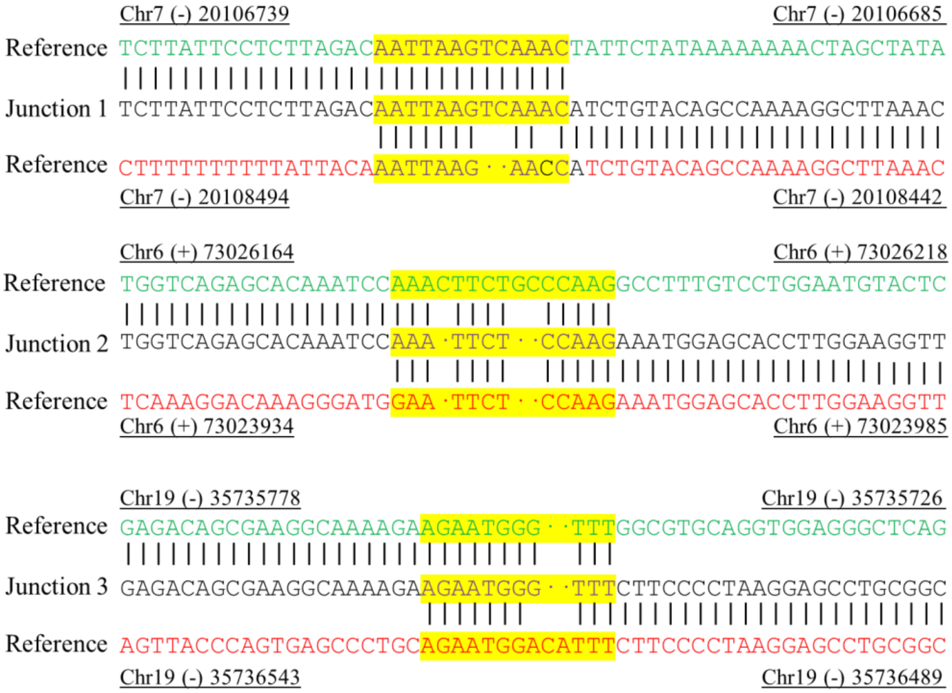

Supplementary Figure S8. Representative junction sequences with 4-16 bp microhomologies between breakpoint ends. Bases highlighted in yellow are the micro-homology sequences.

Supplementary Figure S9.

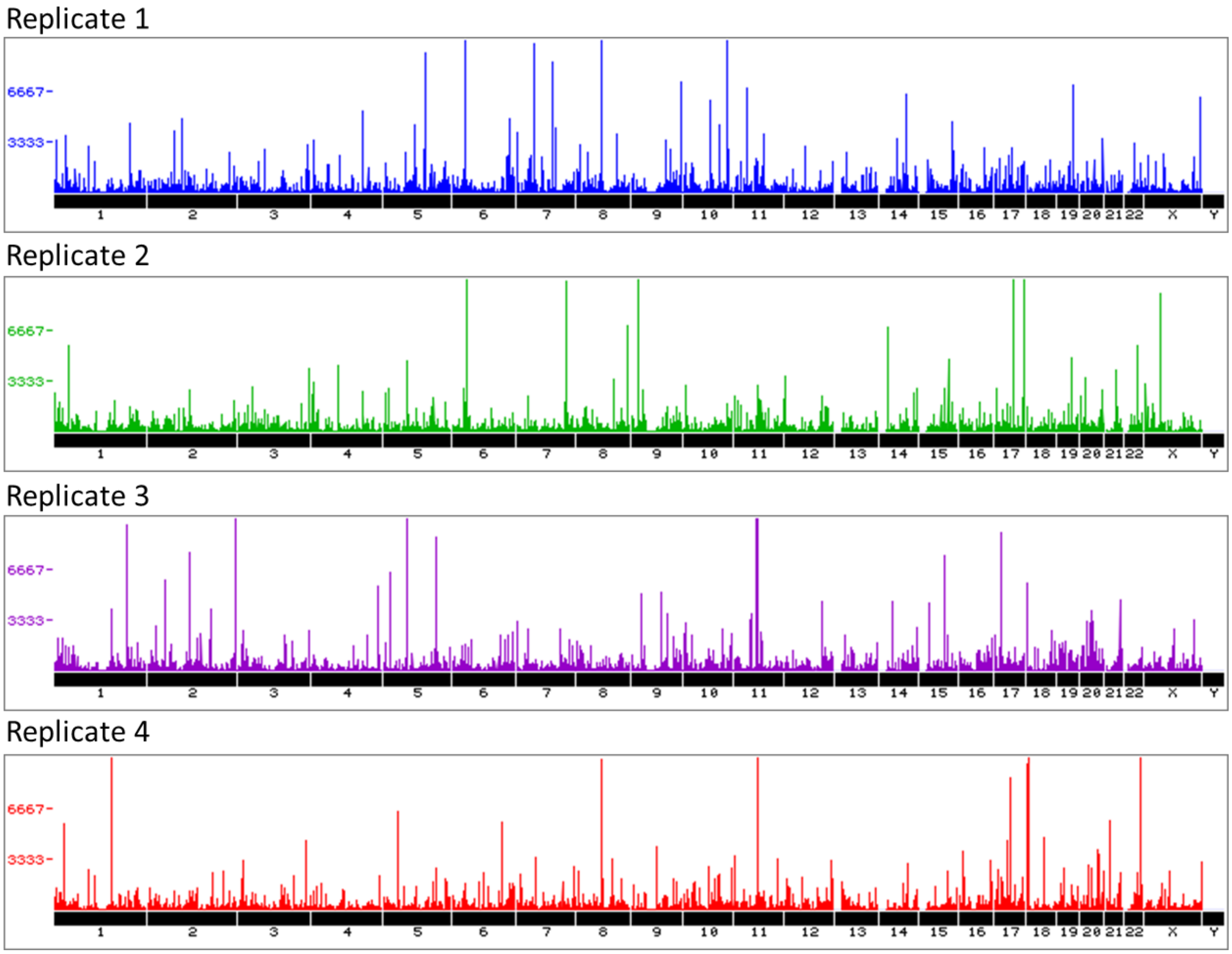

Supplementary Figure S9. Genome view of eccDNA distribution in four replicates of sample 4. The X axis stands for the 23 pairs of chromosomes. The Y axis shows the normalized read depth (normalized to total read count of all split reads with read depth >10).
